# Marine-Derived Natural Lead Compound Disulfide-Linked Dimer Psammaplin A: Biological Activity and Structural Modification

**DOI:** 10.3390/md17070384

**Published:** 2019-06-27

**Authors:** Qinxue Jing, Xu Hu, Yanzi Ma, Jiahui Mu, Weiwei Liu, Fanxing Xu, Zhanlin Li, Jiao Bai, Huiming Hua, Dahong Li

**Affiliations:** 1 Key Laboratory of Structure-Based Drug Design & Discovery, Ministry of Education, School of Traditional Chinese Materia Medica, Shenyang Pharmaceutical University, Shenyang 110016, China; 2 Wuya College of Innovation, Shenyang Pharmaceutical University, Shenyang 110016, China

**Keywords:** psammaplin A, marine natural product, biological activity, structural modification

## Abstract

Marine natural products are considered to be valuable resources that are furnished with diverse chemical structures and various bioactivities. To date, there are seven compounds derived from marine natural products which have been approved as therapeutic drugs by the U.S. Food and Drug Administration. Numerous bromotyrosine derivatives have been isolated as a type of marine natural products. Among them, psammaplin A, including the oxime groups and carbon–sulfur bonds, was the first identified symmetrical bromotyrosine-derived disulfide dimer. It has been found to have a broad bioactive spectrum, especially in terms of antimicrobial and antiproliferative activities. The highest potential indole-derived psammaplin A derivative, UVI5008, is used as an epigenetic modulator with multiple enzyme inhibitory activities. Inspired by these reasons, psammaplin A has gradually become a research focus for pharmacologists and chemists. To the best of our knowledge, there is no systematic review about the biological activity and structural modification of psammaplin A. In this review, the pharmacological effects, total synthesis, and synthesized derivatives of psammaplin A are summarized.

## 1. Introduction

**Figure 1:**
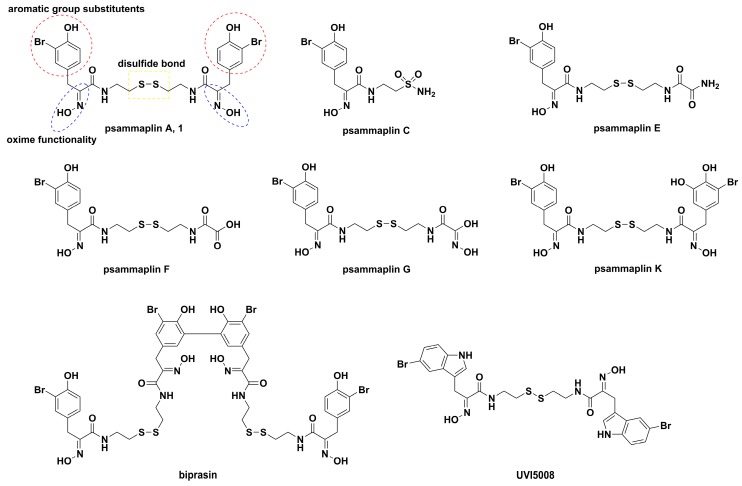
The chemical structure of psammaplin compounds, biprasin and UVI5008.

Accumulating evidence indicates that natural products isolated from plants, animals, and microorganisms have played irreplaceable roles in the development of new drugs for human therapeutics [1,2,3,4,5,6,7,8,9]. It is noteworthy that marine natural products are considered to be extremely valuable resources of natural products and are furnished with diverse chemical structures and various bioactivities [10,11,12]. With the rapid development of technologies of scuba diving and marine prospection, great interest has been shown in unexplored marine natural products, which are considered to be potential sources for drug discovery [13,14,15,16]. To date, seven compounds derived from marine natural products, including cytarabine [17], vidarabine [18], ziconotide [19], omega-3 acid ethyl esters [20], eribulin mesylate [21], brentuximab vedotin [22], and *iota*-carrageenan [17], have been approved as therapeutic drugs by the U.S. Food and Drug Administration. The symmetrical disulfide dimer psammaplin A (**1**, Figure 1), belonging to open-chain α-oximinoamidesis, was originally isolated from *Psammaplysilla* (revised to *Pseudoceratina*) sp. and an unidentified sponge in 1987 [23,24,25], which represents the first isolated natural product containing oxime and disulfide moieties from marine sponge. Subsequently, biprasin, psammaplin C, psammaplin E, psammaplin F, psammaplin G, and psammaplin K (Figure 1) were also obtained [26,27,28,29]. Of these compounds, psammaplin A has attracted much attention because of its strong antimicrobial and cytostatic properties. Psammaplin A displays antibacterial activity mainly against *Staphylococcus aureus* (SA) and methicillin-resistant *Staphylococcus aureus* (MRSA) due to DNA gyrase inhibition and bacterial DNA synthesis arrest [30]. It was also reported that psammaplin A possesses antiproliferative activities against various cancer cell lines, including triple-negative breast (TNBC, MDA-MB-231), doxorubicin-resistant human breast (MCF-7/adr), colon (HCT15), ovarian (SK-OV-3), lung (A549, LM4175), bone (BoM1833), endometria, brain (BrM-2a), skin (SK-MEL-2), and central nervous system (XF498) cancer cell lines [29,31,32,33,34]. Additionally, the bactericidal and cytotoxic effects of psammaplin A were related to multiple enzyme inhibition, such as DNA gyrase [30], topoisomerase II [35], chitinase [36], farnesyl protein transferase [37], mycothiol-*S*-conjugate amidase [38], leucine aminopeptidase [37], DNA polymerase α-primase [39], aminopeptidase N [40], and especially potent inhibitory effects on histone deacetylases (HDAC) and DNA methyltransferase (DNMT) enzymes [28]. These enzymes exert extremely important roles in the epigenetic regulation of gene expression. Moreover, psammaplin A also exhibits potent enzyme inhibitory and antiproliferative activities under reduced conditions in cells, which indicates that psammaplin A could be used as a natural prodrug [41]. Although psammaplin A possesses a broad spectrum of bioactivities, its in-depth study has been hindered due to the limited amount of the compound that can be isolated from marine microorganism sources, as well its poor physiological stability. Inspired by this, many research groups have carried out its total synthesis and synthesis of derivatives. For example, the most potential indole-derived psammaplin A derivative, UVI5008 (Figure 1), was used as an epigenetic modulator with multiple enzyme inhibitory activities [42].

Over the last two decades, more than ten reviews have covered the field of marine natural products from various distinct viewpoints [43,44,45,46,47,48,49,50,51,52,53,54,55,56,57]. Hentschel et al. published an excellent paper on the synthesis of oximinotyrosine-derived marine natural products, including psammaplin A, covering the literature until 2009 [43]. Therefore, the synthetic methods of psammaplin A summarized in this review are those reported after 2009. In addition, structural modification work, structure–activity relationships, and further mechanistic studies of psammaplin A in the field of antibacterial activity and cytotoxicity have been studied by some research groups; these studies consistently confirm that the disulfide bond and the oxime moieties are indispensable for the bioactivities of psammaplin A. However, there is no systematic review of the biological activity and structural modification of psammaplin A. Thus, in this review, the pharmacological effects, total synthesis, and derivatives of psammaplin A are summarized.

## 2. Synthetic Chemistry

Although psammaplin A possesses a broad bioactive spectrum, its in-depth study has been hindered due to the limited amount of the compound that can be isolated from marine microorganism sources. Consequently, many research groups have carried out its total synthesis. To the best of our knowledge, Hentschel et al. have summarized the total synthesis of psammaplin A, covering the literature until 2009 [43]. Therefore, here, we update the total synthesis methods.

**Scheme 1:**
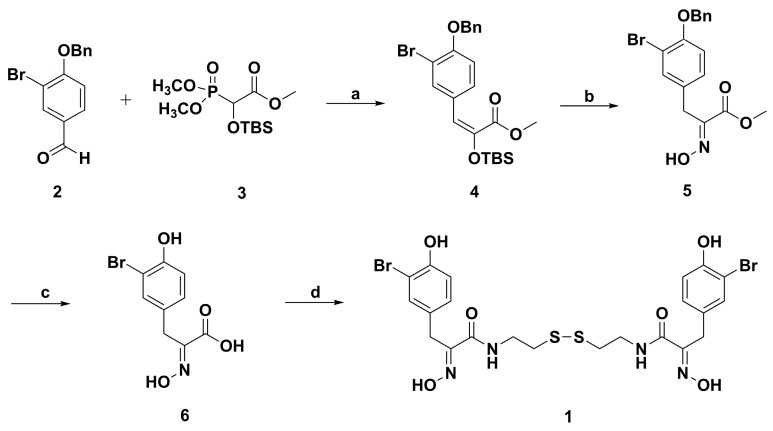
Synthesis route of psammaplin A by the Lindel group. Reagents and conditions: (**a**) LDA, THF, −78 °C, 15 h; (**b**) NEt_3_·3HF, MeOH, rt, 30 min; (**c**) (i) H_2_, Pd/C, dioxane, rt, 41 h; (ii) LiOH·H_2_O, THF/H_2_O, rt, 40 h; (**d**) cystamine dihydrochloride, NHS, DCC, Et_3_N, DMF, rt, 15 h.

Psammaplin A (**1**, Figure 1) consists of a symmetrical disulfide with a cystamine linker functionalized on both sides by tyrosine-derived *α*-(hydroxyimino)acyl moieties [58]. Briefly, the conventional total synthesis method of psammaplin A is initiated from tyrosine or phenylpyruvic acid derivatives [59,60,61,62]. Then, the Lindel group optimized the synthetic method of psammaplin A through the Horner–Wadsworth–Emmons (HWE) route (Scheme 1) [63]. The intermediate **4** was synthesized by the HWE reaction of phosphonate **3** with aldehyde **2**, then oxime **5** was formed after desilylation of **4** under the condition of NEt_3_·3HF. Debenzylation of **5** in the presence of H_2_/Pd–C gave the hydroxyimino isomers; later, methyl ester saponification with lithium hydroxide obtained acid **6**. Finally, the esterification of **6** with two equivalents of cystamine dihydrochloride under the condition of NEt_3_, DCC, and *N*-hydroxysuccinimide (NHS) in DMF generated the natural product psammaplin A.

**Scheme 2:**
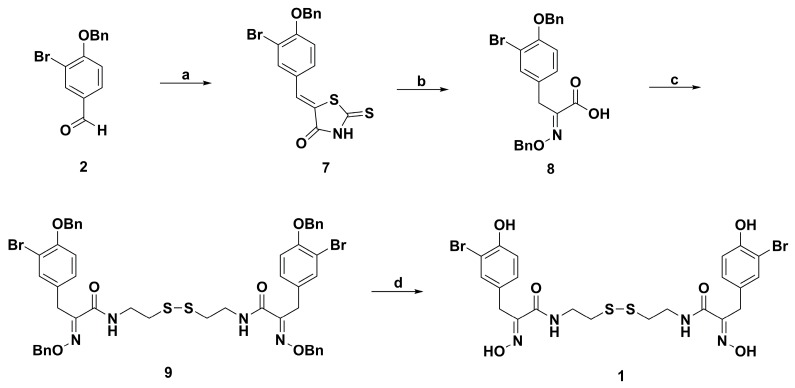
Synthesis route of psammaplin A of the Harburn Group. Reagents and conditions: (**a**) Rhodanine, NH_4_OAc, toluene, reflux; or Rhodanine, NaOAc, AcOH, reflux; (**b**) (i) NaOH, H_2_O; (ii) HCl (10%), 0 °C; (iii) HCl·NH_2_OBn, NaOAc, EtOH, 70 °C; (**c**) cystamine dihydrochloride, EDC, HOBt, Et_3_N, CH_2_Cl_2_; (**d**) TMSI, CH_2_Cl_2_.

The Harburn group reported another synthetic route starting from the benzaldehyde **2** (Scheme 2) [64]. Firstly, **2** was converted to benzylidene rhodanine **7** in excellent yield. Then, hydrolysis, acidification, and oximation of **7** afforded *O*-benzyl protected oximino acid **8**. The coupling of **8** with cystamine generated sponge metabolite **9** protected by the benzyl group. Finally, deprotection of **9** proceeded successfully in CH_2_Cl_2_ using TMSI to provide psammaplin A.

**Scheme 3:**
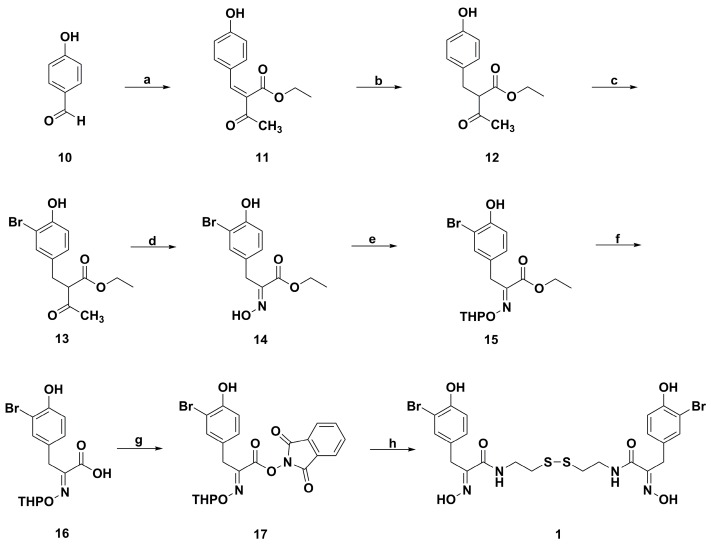
Synthesis route of psammaplin A of the Park group. Reagents and conditions: (**a**) ethyl acetoacetate, piperidine, AcOH; (**b**) H_2_, Pd/C, MeOH; (**c**) KBrO_3_, KBr, 0.5 M-HCl, MeOH; (**d**) *n*-BuONO, AcOEt, NaOEt, EtOH, 0 °C; (**e**) DHP, PTSA, CH_2_Cl_2_; (**f**) 1 N-KOH, EtOH; (**g**) *N*-hydroxyphthalimide, EDC, 1,4-dioxane; (**h**) (i) cystamine, Et_3_N, MeOH, 1,4-dioxane; (ii) 1 N HCl/Et_2_O CH_2_Cl_2_/MeOH.

The Park group developed a new efficient and concise synthetic method for preparing psammaplin A (Scheme 3) [65,66]. Initially, the α,β-unsaturated ester **11** was obtained by Knoevenagel condensation of 4-hydroxybenzaldehyde (**10**) with ethyl acetoacetate using acetic acid and piperidine. Then the catalytic hydrogenation of **11** generated compound **12** through Pd/C and H_2_ in methanol. The bromination of **12** with KBrO_3_ and KBr in methanol under the condition of 0.5 M-HCl provided **13**. The treatment of *n*-butylnitrite with **13** in ethanol under the condition of sodium ethoxide at 0 °C yielded *α*-NO substituted **13** analogue. After *α*-nitrosation, the fragmentation initiated by addition of ethoxide anion to the acetyl group led to ethyl acetate release, followed by rearrangement to obtain the α-oxime ester **14**. The oxime hydroxyl group of **14** was protected with dihydropyran (DHP) under the condition of catalytic amount *p*-toluenesulfonic acid (PTSA) to obtain **15**. Then, **15** was hydrolyzed to the acid **16** with 1 N potassium hydroxide in ethanol. **16** and *N*-hydroxyphthalimide were coupled under the condition of EDC in 1,4-dioxane to synthesize **17**, followed by adding cystamine to afford the THP-protected psammaplin A. Finally, the THP-protected psammaplin A was deprotected in the presence of the solution of methanolic hydrochloride to get psammaplin A.

## 3. Pharmacological Activity

The pharmacological activities of psammaplin A and its derivatives include antibacterial, antiviral, anticancer, insecticidal, embryo development promotive, chemical defensive eryptosis inducing, and anticancer activities. These works can be classified as follows.

### 3.1. Antimicrobial Activity

**Figure 2:**
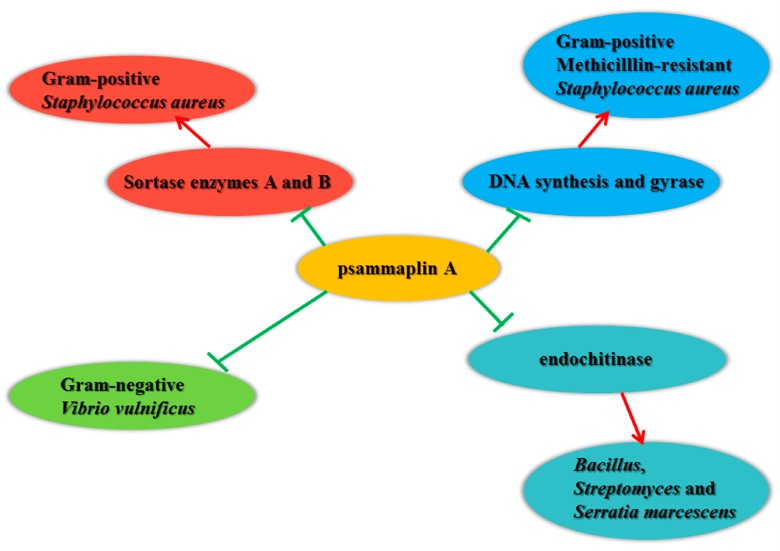
Schematic representation of the antimicrobial activity of psammaplin A.

Sortase enzymes, transpeptidases from Gram-positive bacteria, are responsible for anchoring surface protein virulence factors to the peptidoglycan cell wall layer. In Gram-positive SA, sortase isoform deletion results in significant reduction in infection potential and virulence. Psammaplin A showed potent inhibition of Sortase A and B, and adhesion of SA cells to fibronectin (Figure 2) [67].

The threat of multidrug-resistant bacterial strains against human health is increasing, and a large number of people die every year from the spread of resistant strains. Studies by Kim et al. showed that psammaplin A could inhibit Gram-positive bacteria, such as MRSA. Psammaplin A inhibited DNA synthesis with an IC_50_ value of 2.83 μg/mL, and DNA gyrase activity with an IC_50_ value of 100 μg/mL. [30]. Franci et al. screened a group of previously identified epigenetic regulators, and some could change the growth of Gram-positive bacteria. UVI5008 (Figure 1), a derivative of psammaplin A, was identified. The growth inhibition activity against MRSA was caused by cell wall modification [68].

Lee et al. measured the inhibition of Gram-negative *Vibrio vulnificus* (*V. vulnificus*)-induced cytotoxicity by 12 compounds from natural seafood in intestinal epithelial cells (INT-407). The results showed that psammaplin A significantly inhibited *V. vulnificus*-induced cytotoxicity, which indicated that psammaplin A could be developed for the prevention and treatment of *V. vulnificus* infection [69]. 

Tabudravu et al. tested the chitinase inhibition activity of psammaplin A in *Bacillus*, *Streptomyces*, and *Serratia marcescens*. The results showed that psammaplin A noncompetitively inhibited endochitinase activity with IC_50_ values of ranging from 50 to 100 μM [36].

### 3.2. Antiviral Activity

As a chronic infectious disease, hepatitis C can cause liver cancer; NS3 nucleoside triphosphatase (NTPase)/helicase plays an important role in hepatitis C virus (HCV) replication. Salam et al. screened inhibitors of NS3 helicase from marine organism extracts by a photo-induced electron transfer (PET) system. Psammaplin A showed the apparent Km value of 0.4 μM of NS3 ATPase activity which indicated no influence. The inhibitory effect of psammaplin A on viral replication was verified by experiments and it can be used as a potential antiviral agent [70]. Psammaplin A also shows anti-HIV activity. Richard et al. reported that psammaplin A could induce the expression of latent HIV-1 provirus in Jurkat full-length T cell lines (clones 8.4, 9.2, and 10.6). Psammaplin A synergistically enhanced the expression of HIV-1 when combined with the protein kinase C (PKC) activator prostratin, but not the histone deacetylase inhibitor (HDACi) panobinostat, indicating its latency to be a reversing agent (LRA) to induce proviral expression [71].

### 3.3. Embryo Development Promotive Activity

Reprogramming of donor somatic nuclei to an omnipotent embryonic state is a major obstacle to successful cloning, and treatment of cloned embryos with epigenetic modifiers such as HDACi can improve cloning efficiency. Acting as a novel HDACi, the effects of psammaplin A on the development and quality of cloned mouse embryos were investigated by Mallol et al. The experimental results confirmed that psammaplin A increased the cloning efficiency of mice more than valproic acid (VPA) [72].

For the development of somatic cell nuclear transfer (SCNT) embryos, the embryonic stem cell (ESC)-derived rates from the obtained blastocysts and intracytoplasmic sperm injection (ICSI) fertilized embryos were determined with or without treatment of psammaplin A. The results showed that psammaplin A-treated SCNT exerted increased nuclear transfer ESC derivation and blastocyst rates, and further increased embryo delay, which was not necessarily related to the epigenetic effects [73].

### 3.4. Insecticidal Activity

Chitinase is an interesting target that interferes with growth and develops alternative strategies for controlling pests. Psammaplin A is a chitinase inhibitor and acts as an effective active ingredient for the termite bait program. Husen et al. evaluated the impact of psammaplin A on *Reticulitermes flavipes* (*R. flavipes*) by using a no-choice feeding bioassay of eastern underground termites. The results indicated that chitinase inhibitor psammaplin A was toxic to *R. flavipes* and induced mortality in a non-concentration-dependent manner [74]. After that, Husen et al. further designed a trial to evaluate the palatability, feeding deterrence, consumption, and subsequent mortality. Psammaplin A was incorporated into filter paper diets and the treated filter papers were used as food source or bait for termite workers used in this study. In the no-selective feeding trial, the diet consumption of termites fed a 0.3% (2–5 weeks) and 0.15% (4–5 weeks) psammaplin A treatment diet was significantly reduced. In the double selection test, termites consumed almost the same amount of diet treated with psammaplin A as an untreated diet (except for diets treated with 0.3% psammaplin A). Additionally, in the no-select bioassay, termite mortality from diets treated with chitinase inhibitors was significantly higher than in untreated diets; at the same time, the biological activity of psammaplin A-treated diets in the double-select feeding arenas was reduced by more than 50%. These results indicate that chitinase inhibitors have new potential [75].

Psammaplin A can also be used as an aphid management tool. In a previous study, Saguez et al. reported the aphicidal effects of psammaplin A. Psammaplin A reduced fecundity, increased larval mortality, and reduced body size. An artificial diet was used to provide *M. persicae* with active (1, 10, 100 and 500 μg/mL) and inactive (500 μg/mL) bacterial (*Serratia marcescens*) chitinase. These compounds increased the nymphal viability at all active chitinase doses compared to the control diet, whereas inactive chitinase cannot [76]. Afterward, four chitinase inhibitors, cyclo-(histidine-valine), cyclo-(valine-tyrosine), psammaplin A, and allosamidin, were selected for feeding experiments with *M. persicae* (Sulzer), the peach-potato aphid. Artificial feed was used to supply 10, 50, and 100 μg/mL. The results showed that psammaplin A was the most toxic compound, increasing the mortality of all aphids at 50 and 100 μg/mL [77,78]. 

### 3.5. Active Chemical Defense

Active chemical defense, which rapidly transforms precursor molecules of defensive compounds after tissue damage, is widely found in terrestrial and marine plants, but is extremely rare in marine invertebrates. Thoms et al. observed that wound activation converted psammaplin A sulfate to psammaplin A in the tissue of the tropical sponge *Aplysinella rhax*. The same group, in a feeding test of the puffer fish *Canthigaster solandri*, showed that the antifeeding activity of psammaplin A was increased compared with the sulfate, which suggested that psammaplin A possessed defensive activity. A series of observations on their response to other sponge species indicated that marine organisms may have more active defenses [79].

### 3.6. Eryptosis Induction Acitivity

The cellular mechanisms that stimulate eryptosis include oxidative stress, cytosolic Ca^2+^ activity ([Ca^2+^]_i_), and increased ceramide; Abdulla Al Mamun Bhuyan et al. found that psammaplin A increased ceramide abundance and dichlorodihydrofluorescein diacetate (DCFDA) fluorescence and triggered cell shrinkage and phospholipid scrambling of the erythrocyte cell membrane, caused by induction of oxidative stress, increase of [Ca^2+^]_i_, and enhanced appearance of ceramide [80].

### 3.7. Anticancer Effects

**Figure 3:**
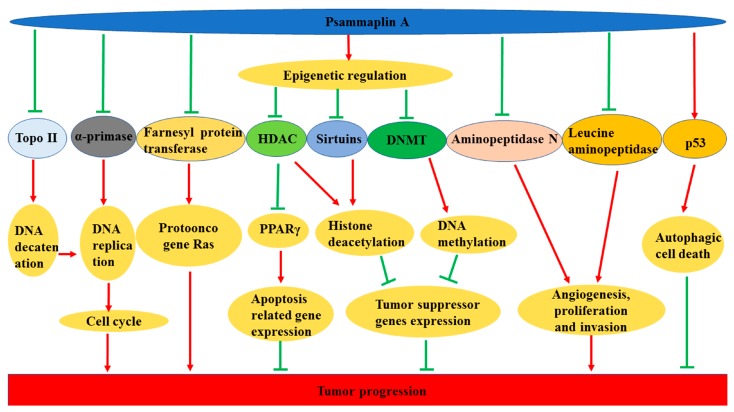
Schematic representation of the cytotoxic effects of psammaplin A.

The underlying anticancer properties of psammaplin A may depend on its efficacy in regulating enzymes that regulate apoptosis, differentiation, invasion, proliferation, angiogenesis, and DNA replication and transcription (Figure 3). Jiang et al. found that psammaplin A showed significant cytotoxic activity against the RAW264.7 cell line and could substantially inhibit SV40 DNA replication. The polymerase α-primase was one of the main targets [39]. Psammaplin A radiosensitization of glioblastoma U-373MG and lung cancer A549 cell lines might be due to the inhibition of DNA repair [81]. Godert et al. found that psammaplin A was a potent DNMT inhibitor in vitro, but it failed to alter the level of genomic DNA methylation in treated human colon carcinoma HCT116 cells [59]. Psammaplin A also inhibited cell growth of lung cancer NCI-H226 Bap1 null cells at a concentration between 1/10,000 μL and 1/1000 μL, while exhibiting minimal toxicity to human neuroblastomal SKN cells. When CPT was added, the performance strongly indicated that psammaplin A could exert a synergetic DNA damaging effect [82]. Kim et al. reported the tissue distribution and pharmacokinetics of psammaplin A as a DNMT and HDAC inhibitor in mice. The intravenous injection dose was 10 mg/kg and psammaplin A was rapidly eliminated, with the average half-life of 9.9 min and the systemic clearance (CLs) was 925.1 ± 570.1 mL/min. Psammaplin A was highly distributed in lung tissues, with lung-to-serum partition coefficients (Kp) of 49.9 to 60.2, while the concentrations in other tissues were either comparable to or less than serum concentrations [83].

Epigenetic dysregulation is one of the causes of cancer, and epigenetic factors are condidered as therapeutic targets. Nebbioso et al. pointed out that UVI5008 was an epigenetic modifier that inhibited HDAC, DNMT, and sirtuins, which efficiently induced selective death of cancer cells and exerted its activity in genetic mouse models of human breast cancer and several human tumor xenografts. Its anticancer activity involved the activation of reactive oxygen species and death receptors. UVI5008 showed strong anticancer properties with IC_50_ values from 0.2 to 3.1 μM. It also showed activity in vivo in HCT116- or MCF-7-xenografted mice (40 mg/kg) and ex vivo in acute myeloid leukemia (AML) blasts (5 μM) [84].

Massague et al. found that psammaplin-based HDAC inhibitors differentially induced hypoxia-inducible factor 1 (hif-1) activation, inhibited HDAC activity, and disrupted the growth of organic metastatic TNBC subclones. The results showed that psammaplins significantly inhibited the growth of bone (BoM1833) tumor spheres in the 3D culture system [31]. Peroxisome proliferator-activated receptors (PPARs) are ligand-activated transcription factors which have been shown to inhibit the growth of human breast tumor cells, induce apoptosis, and promote terminal differentiation. Psammaplin A can activate PPARγ and induces apoptosis in MCF-7 cells [85].

Psammaplin A showed inhibitory activity in enzyme assays and antiproliferation assays with IC_50_ values of 0.003 and 1 μM, respectively. It selectively induced high acetylation of histones, resulting in upregulation of the well-known HDAC target gene gelsolin at the transcriptional level. Furthermore, the reduced psammaplin A showed stronger inhibitory activity than the unreduced one. It is noteworthy that glutathione-depleted cells were not sensitive to psammaplin A, which indicated that cellular reduction was responsible for HDAC inhibition [41]. Baud et al. also reported the cytotoxicity and enzyme inhibitory activity against recombinant HDAC1 by the active monomer (thiol) form of psammaplin A [86]. DNMT1 inhibitory activity was not found in another study [60].

In addition, psammaplin A inhibits aminopeptidase N (APN), which plays an important role in tumor progression and is involved in processes such as proliferation, adhesion, angiogenesis, and tumor invasion [40]. Psammaplin A also has the ability to inhibit mycothiol-*S*-conjugate amidase (MCA) [38], topoisomerase II [35], farnesyl protein transferase [37], and leucine aminopeptidase [37]. In addition, psammaplin C is a natural product of primary sulfonamide. Mujumdar et al. evaluated its inhibitory properties for the treatment-related carbonic anhydrase (CA) zinc metalloenzyme. At the same time, the analog psammaplin C showed unprecedented inhibition levels with a K_i_ of 0.79 nM of isoenzyme hCA XII. They also proposed the protein X-ray crystal structure of psammaplin C complexed with human CA and determined the binding posture with the hCA II, hCA IX, and hCA XII mimetic active sites [26]. Psammaplin C also reduced the efflux of temozolomide by P-glycoprotein and resensitizes the primary neurosphere to temozolomide. Salaroglio et al. revealed that the interaction of CA XII and Pgp could ultimately block the efflux function of Pgp to improve the prognosis of patients with glioblastoma [87].

Targeting the autophagic pathway plays a key role in chemotherapeutic approaches to treat human cancers and preventing tumor-derived chemoresistance; at the same time, some marine-derived compounds show this potency. Ratovitski et al. used psammaplin A to induce the expression of several autophagic signaling intermediates in human glioblastoma, squamous cell carcinoma, and colorectal cancer cells through transcriptional regulation by tumor protein p53 family members [34].

Psammaplin A also shows anticancer activity by inducing cell cycle arrest and apoptosis. Kim et al. reported that psammaplin A could significantly inhibit the proliferation of MCF-7/adr cells in a dose-dependent manner, and the cells arrested at the G_2_/M phase [33]. Ahn et al. investigated the antitumor effect on Ishikawa endometrial human cancer cells. The results showed that psammaplin A could significantly inhibit Ishikawa cell proliferation in a dose-dependent manner. Psammaplin A significantly induced the expression of acetylated H3 and H4 histones, resulted in significant apoptosis associated with p53-independent p21WAF1 expression, and showed antiproliferative effects by selectively inducing genes involved cell cycle arrest [32].

## 4. Medicinal Chemistry

Inspired by the unique symmetrical structure of bromotyrosine-derived disulfide dimer scaffolds, a collection of derivatives was constructed and synthesized through structural modifications, aiming to explore potential therapeutic value and study the structure–activity relationships. With this consideration in mind, we reviewed the derivatives of psammaplin A (Figure 1) as follows, according to their different activities.

### 4.1. Antibacterial Derivatives

**Figure 4:**
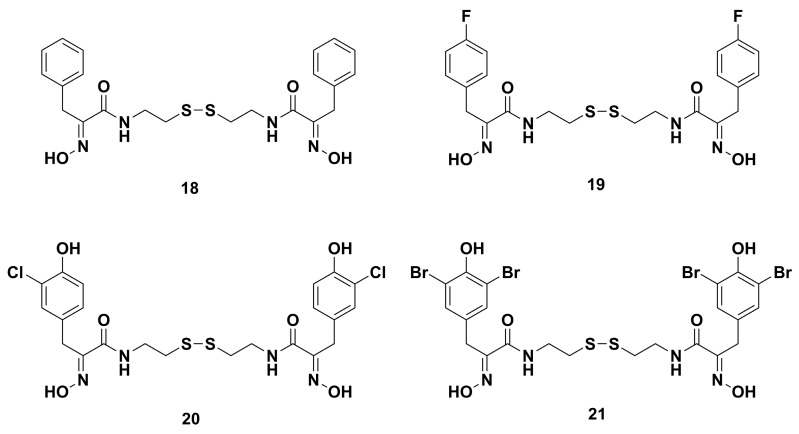
Chemical structures of the synthetic homodimeric derivatives **18**‒**21**.

**Figure 5:**
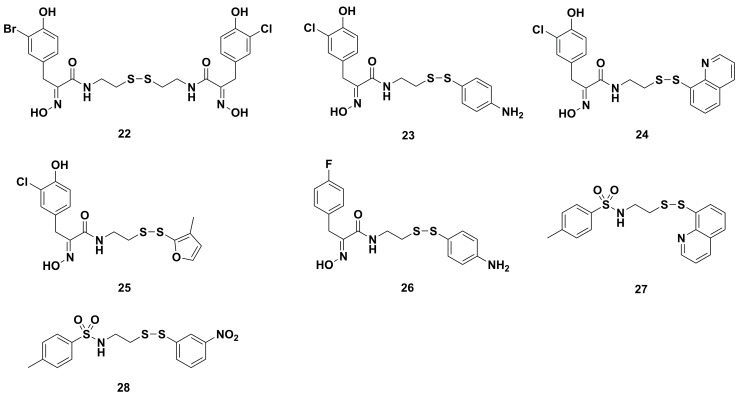
Chemical structures of three types of psammaplin A derivatives **22**‒**28**.

Some homodimeric and heterodimeric analogues of psammaplin A were refined by Nicolaou and coworkers utilizing combinatorial chemistry through a disulfide exchange strategy [61]. Combinatorial chemistry techniques have played an important role in the synthesis and structural activity optimization of bioactive natural products [88]. Among the synthetic homodimeric derivatives, compounds **18**, **19**, **20**, and **21** (Figure 4) showed significant antibacterial effects against MRSA. Moreover, the heterodimeric derivatives fell into three types (A–C) (Figure 5). Type A representative compound **22** consisted of two similar psammaplin-like structures. Type B representative compounds **23**–**26** were comprised of one psammaplin-like component conjugated to an alkyl or aryl group. Type C representative compounds **27** and **28** had no resemblance to the original psammaplin A structure. Among these heterodimeric derivatives, compounds **23**–**28**, with MIC values of 1.22, 2.43, 1.61, 3.90, 4.10, and 3.14 μg/mL, respectively, possessed higher antibacterial activity than psammaplin A (MIC 5.47 μg/mL). Especially, compound **23** showed similar activity to clinically used drugs vancomycin and ciprofloxacin, with MIC values of 0.83 and 0.89 μg/mL, respectively.

**Figure 6:**
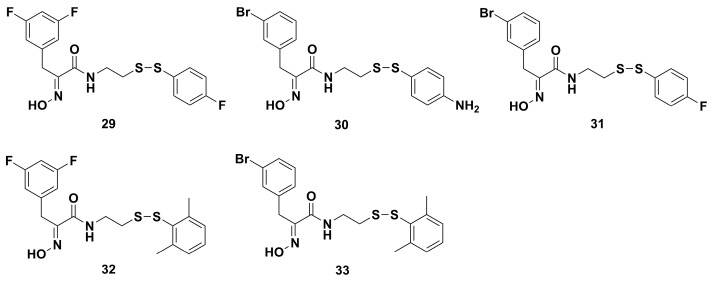
Chemical structures of the representative heterodimeric derivatives **29**‒**33**.

In the same year, in order to obtain better antimicrobial agents, Nicolaou and coworkers continued to optimize the heterodimeric lead compounds **23**–**26**, mainly by studying the toxicity, potency, and nonspecific protein-binding effects through molecular design, structural modification, and mechanism of action [89]. Subsequently, a series of highly potent heterodimeric derivatives were afforded by parallel synthesis. Some representative compounds **29**–**33** (Figure 6) possessed 50-fold higher activities than their parent against both SA and MRSA. The average MIC values of compounds **30**–**33** were 0.09, 0.12, 0.29, and 0.12 μg/mL against SA, respectively, and 0.09, 0.11, 0.27, and 0.11 μg/mL against MRSA, respectively. In an in vitro toxicity assay, a therapeutic index (TI) ratio, as an estimate of the selectivity of the heterodimeric derivatives afforded the average IC_50_ value of a compound against fibroblast and lymphocyte cells, and was divided by the average MIC value against SA and MRSA strains, and the results showed that heterodimer **31**, comprised of 3-bromo-phenyl alaine and 4’-fluorophenyl groups, had a good TI of 37.5, which indicated low toxicity and good selectivity. The mechanism study failed to confirm the reported inhibition of DNA gyrase [30] by psammaplin A and its derivatives. However, their studies also propose a nonspecific redox-based mechanism for these heterodimers.

**Scheme 4:**
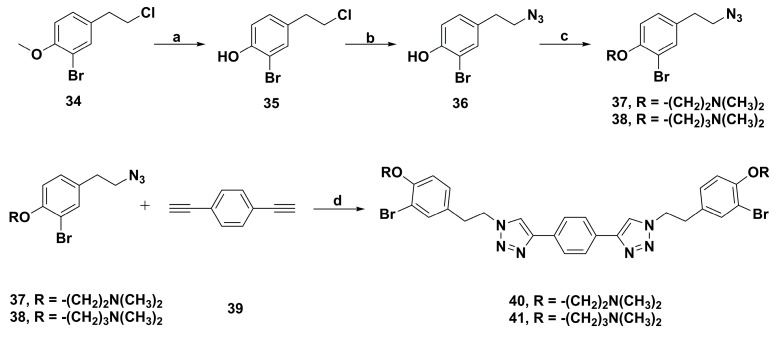
Synthesis of psammaplin A bis-triazole derivatives **40** and **41**. Reagents and conditions: (**a**) BBr_3_/CH_2_Cl_2_, 0 °C, rt, 4 h; (**b**) NaN_3_/DMF, 5 h, 90 °C; (**c**) K_2_CO_3_, 18-crown-6, 2-dimethylaminoethyl chloride or 3-chloro-*N*,*N*-dimethylpropan-1-amine, acetone, reflux; (**d**) CuSO_4_/sodium ascorbate, DMF/H_2_O (2:1), 24 h, rt.

Recently, Blache et al. synthetized a series of psammaplin A analogues using click chemistry based on a framework of bis-triazole (Scheme 4) [90]. Among them, the representative derivatives of the dimethylaminoethyl chain **40** and the dimethylaminopropyl chain **41** possessed potent antibiofilm activity against three Gram-negative strains, including *Pseudoalteromonas lipolytica* (TC8), *Paracoccus* sp. Strain (4 M6), and *Pseudoalteromonas ulvae* (TC14), with EC_50_ close to tributyltin oxide and ampicillin. Furthermore, compounds **40** and **41** were not lethal to bacteria at low concentrations and showed weak bactericidal effects at high concentrations, which indicated they might be used as coantibiotics or nontoxic cobiocides.

### 4.2. Anticancer Derivatives

**Scheme 5:**
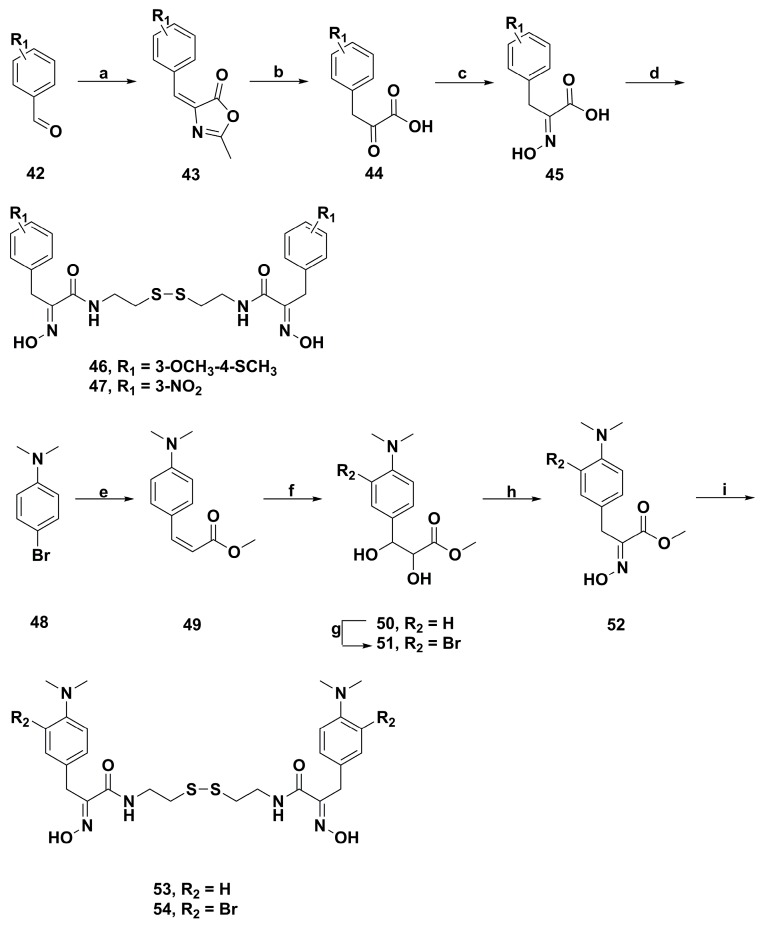
Synthesis of psammaplin A analogues **46**, **47**, **53**, and **54**. Reagents and conditions: (**a**) NaOAc, *N*-Ac-Gly, Ac_2_O; (**b**) HCl; (**c**) HONH_2_·HCl, pyridine; (**d**) EDCI, *N*-hydroxy-succinamide dioxane; nucleophile, NEt_3_, dioxane/MeOH; HCl, CH_2_Cl_2_/Et_2_O/MeOH; (**e**) Pd(OAc)_2_, P(*o*-Tol)_3_, NEt_3_, DMF; (**f**) osmium(VIII) oxide, NMO, MeCN/water; (**g**) NBS, DMF; (**h**) *p*-TsOH·H_2_O, benzene; HONH_2_·HCl, NaOAc, MeOH; (**i**) cystamine, AlMe_3_, CH_2_Cl_2_.

A collection of more than 70 psammaplin A analogues were synthesized by Fuchter and coworkers [91]. The enzyme inhibitory activities against histone deacetylase 1 (HDAC 1) and HDAC 6 were evaluated. The derivatives **46**, **47**, **53**, and **54** (Scheme 5) showed more potent activity than psammaplin A and current inhibitors including trichostatin A and SAHA. Moreover, these compounds also displayed good selectivity for HDAC 1 over HDAC 6. In short, the structure−activity relationship indicated that the derivatives with the electron withdrawing group or the electron donating group in the benzene ring exhibited higher enzyme inhibitory activity than psammaplin A.

**Figure 7:**
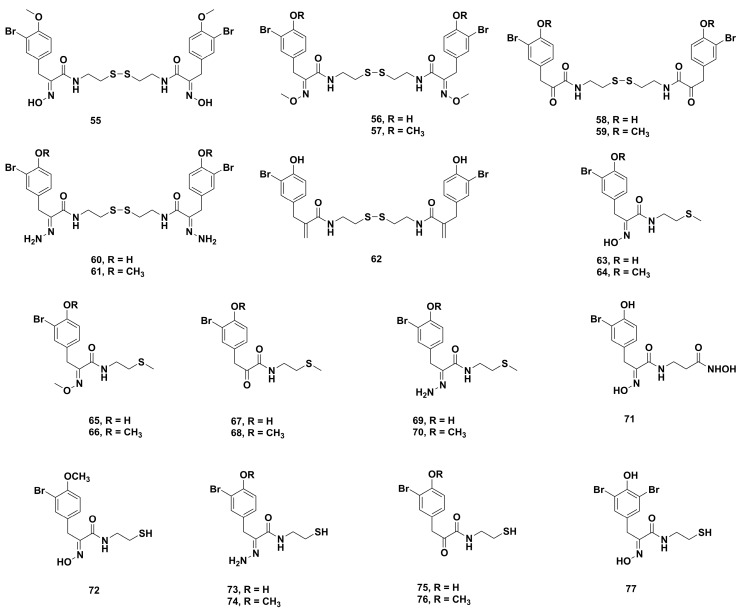
Chemical structures of the psammaplin A derivatives **55**‒**77**.

Subsequently, this group synthesized a library of psammaplin A derivatives (Figure 7) by modifying the disulfide bond, the aromatic group substituents, and the oxime functionality, aiming to study the enzymatic selectivity and mechanism of action against DNA methyltransferases and histone deacetylases [92]. The HDAC assays showed that the disulfide analogues **55**–**62** were less potent than their reduced products containing the free thiol. When the sulfur end group was protected, analogues **63**–**70** showed low to no inhibition of both HDAC 1 and HDAC 6. However, hydroxamic derivative **71** possessed highly potent activities against HDAC 1 (2 nM) and HDAC 6 (190 nM). Among the derivatives changed by the oxime functionality, the oxime-containing analogue **72** and hydrazone analogues **73** and **74** were 444−611 and 80−183-fold more potent, respectively, than the α-ketoamide-containing compounds **75** and **76** against HDAC 1. In the derivatives of aromatic group substituents, compound **77** exhibited the highest selectivity against HDAC 1. However, its potency was minor compared to the presence of the oxime and the free thiol. Unfortunately, psammaplin A and its derivatives were found to have weak inhibitory effects against DNA methyltransferases. On the other hand, the cytotoxicity studies showed that compound **72** had the smallest IC_50_ values of 0.16 and 0.61 μM against human lung carcinoma A549 and human breast carcinoma MCF-7, respectively. Amazingly, **73** was particularly selective against MCF-7 (IC_50_ 3.42 μM) with a ten-fold increase compared to the other cancer cell lines. Besides, hydroxamic acid **55** showed significant antiproliferative activity against A549 and MCF-7, which indicated that the cytotoxicity of derivatives might be related to HDAC inhibition. Western blot analysis showed that treatment with compound **71** could upregulate histone acetylation levels and not affect the tubulin acetylation levels. According to docking studies with HDAC 1, psammaplin A and its derivatives could bind to Zn^2+^.

**Figure 8:**
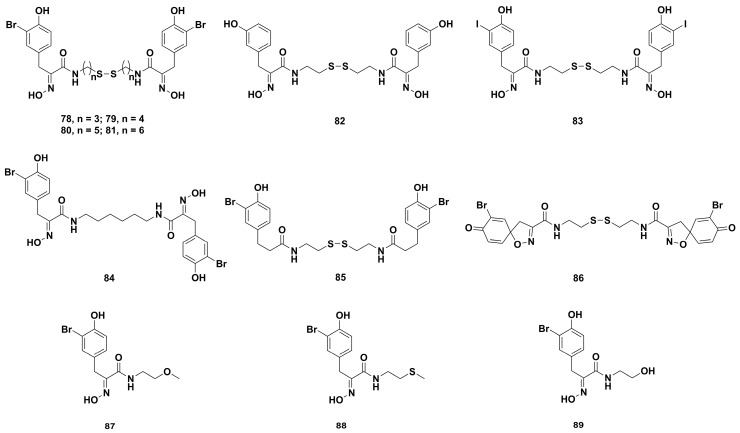
Chemical structures of the psammaplin A derivatives **78**‒**89**.

In order to focus on the structure–activity relationship of the antiproliferative activities of psammaplin A derivatives, de Lera and coworkers synthesized five series of derivatives (Figure 8) by modifications in the halo-tyrosine aryl ring, the oxime bond, the connecting chain length, and the disulfide unit [60]. Subsequently, the HDAC inhibition tests showed that the derivatives **82** and **83** substituted on aryl rings possessed more potent inhibitory activity than psammaplin A. However, compounds **84**–**89** showed no apparent inhibitory effect, which suggested the oxime and disulfide bonds were indispensable for the HDAC inhibition activity. The cell-based assays on the U937 myeloid leukemia cells indicated that derivatives **85**, **82**, and **83** lacking the oxime could cause cell cycle arrest at G_1_ phase, and the spirocycle derivative **86** lacking the free oxime and flexibility could induce apoptosis and arrest cell cycle at the G_2_ phase. The homologues **78**–**81**, including three to six methylene units, and dimer **84**, containing an ethylene group replacing the disulfide bond, had weak apoptotic effects against the U937 cells. Mechanically, compounds **78**–**81**, **84**, **86**, **87**, and **89** could decrease the expression levels of p21WAF1 and tubulin acetylation. The derivative **82** could upregulate the levels of p21WAF1 even higher than SAHA. Moreover, **82** and **83** could increase the acetylated histone H3 levels.

**Scheme 6:**
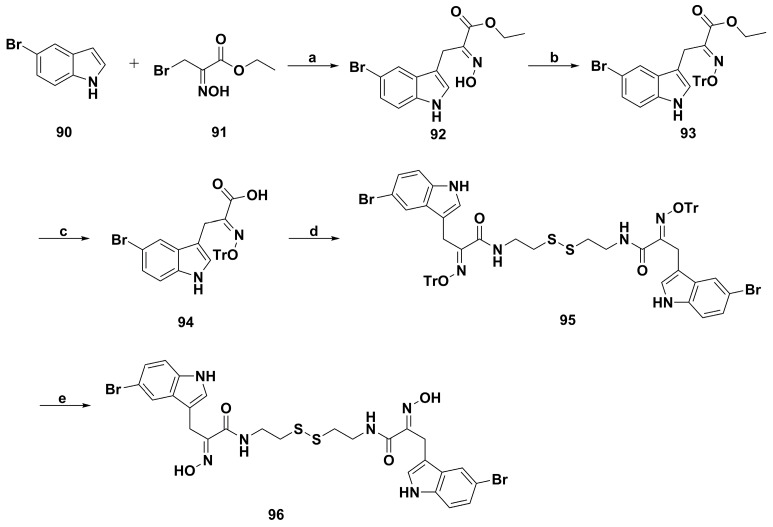
Synthesis of indole-based psammaplin A derivative **96**. Reagents and conditions: (**a**) K_2_CO_3_, CH_2_Cl_2_, 25 °C, 20 h; (**b**) K_2_CO_3_, CH_2_Cl_2_, TrCl, 25 °C, 20 h; (**c**) LiOH·H_2_O, THF/H_2_O (1:1), 25 °C, 20 h; (**d**) cystamine, EDC, NHS, dioxane, 25 °C, 2 h; (**e**) 1 M HCl in Et_2_O, CH_2_Cl_2_, 25 °C, 2 h.

With continued research by de Lera and coworkers [42], a number of indole-based psammaplin A derivatives were designed and synthesized by replacing the *o*-bromophenol group with an indole ring to pursue more potent molecules for epigenetic disorder modulation, such as cancer. Especially, the induction ability of U937 acute myeloid leukemia (AML) cell apoptosis by compound **96** (Scheme 6) was stronger than that of the parent. Cell-based assays affirmed that the presence of disulfide bridge from **96** is essential for cell cycle arrest, differentiation, and induction of apoptosis. Besides, **96** could more efficiently induce α-tubulin acetylation as a sign of HDAC6 inhibition and increase the expression of histone H3 and p21 protein. Derivative **96** also induced cell cycle arrest and apoptosis in ex vivo AML patient blasts. In enzyme-based assays, **96** not only possessed the stronger inhibitory activities against HDAC and DNMT enzymes, but also inhibited the NAD^+^-dependent SIRT deacetylase enzymes. In vivo pharmacokinetics study showed that **96**, as a prodrug, could immediately transform into the glutathione intermediate to exert multiple enzyme inhibitory activities [77]. More importantly, the maximum tolerated dose of **96** was higher than that of listed HDAC inhibitors, which merited further investigation for cancer therapy.

**Figure 9:**
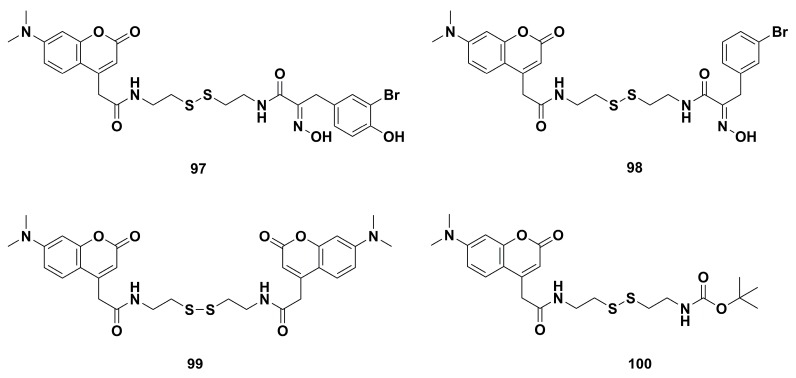
Chemical structures of the psammaplin A fluorescent derivatives **97**‒**100**.

Some psammaplin A fluorescent analogs were synthesized by Lindel and coworkers [63]. The cytotoxicity studies showed the fluorescent 4-coumarinacetyl-α-(hydroxyimino)acyl derivatives **97** and **98** (Figure 9) with IC_50_ values of 0.93 and 1.10 μg/mL, respectively, were about two-fold stronger than that of psammaplin A (IC_50_ 0.42 μg/mL) against the mouse fibroblast L-929 cells. Furthermore, bis- and mono (coumarinyl) derivatives **99** and **100** (Figure 9) lacking α-(hydroxyimino)acyl moieties were not cytotoxic. The HDAC inhibitory activities of the coumarin–psammaplin hybrid **97** (IC_50_ 0.011 μM) was two-fold more potent than psammaplin A (IC_50_ 0.028 μM) by a fluorometric HDAC assay. Afterwards, fluorescence microscopy revealed that compounds **97** and **98** lacking α-(hydroxyimino)acyl units were taken up into the cytoplasm, leading to fluorescence in the nuclear envelope, not in the nucleus, which indicated the disulfide bonds were reduced to the thiol before the disulfide penetrated the nucleus [93]. 

**Scheme 7:**
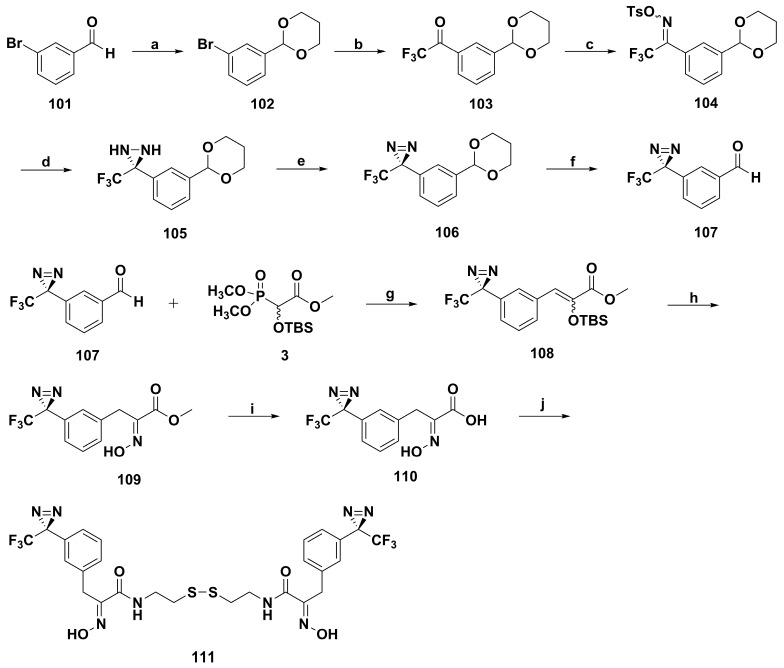
Synthesis of photopsammaplin **111**. Reagents and conditions: (**a**) propane-1,3-diol, *p*-TsOH, toluene, rt, 2.5 h; (**b**) Mg, THF, rt, 2 h; then 0 °C, 2,2,2-trifluoro-1-(piperidin-1-yl)ethanone, 2 h, rt; (**c**) (i) HONH_2_·HCl, pyridine, rt, 9 h; (ii) NEt_3_, *p*-TsCl, 24 h, 0 °C to rt; (**d**) NH_3_, 6 h, −78 °C to rt; (**e**) NEt_3_, I_2_, 15 min, 0 °C, then 3 h, rt, avoiding daylight; (**f**) 0.5 M H_2_SO_4_, acetone/H_2_O, rt, 12 h, quant; (**g**) LDA, THF, −78 °C, 14 h; (**h**) (i) NEt_3_·3HF, MeOH, rt, 2 h; (ii) HONH_2_·HCl, MeOH, rt, 17 h, quant; (**i**) LiOH·H_2_O, THF/H_2_O, rt, 20 h, quant; (**j**) cystamine dihydrochloride, NHS, DCC, NEt_3_, DMF, rt, 15 d.

Soon afterwards, Lindel and coworkers synthesized the first photoreactive psammaplin A derivative **111** by adding 1-azi-2,2,2-trifluoroethyl moieties to benzene rings (Scheme 7) [94]. Photopsammaplin **111** showed antiproliferative activity in vitro with an average IC_50_ value of 1.4 µM against 42 human cancer cell lines, which was especially sensitive to lung cancer (LXFA 526), melanoma (MEXF 276), mammary cancer (MAXF 401 and MCF-7), and bladder cancer (T-24), with IC_50_ values below 0.6 µM. Furthermore, the fluorometric HDAC assay showed **111** was also a potent HDAC inhibitor, with an IC_50_ value of 35 nM. With this information, photopsammaplin **111** might be considered as a good candidate of photoaffinity labeling, which played an important role in new targets identification of psammaplin A.

**Scheme 8:**
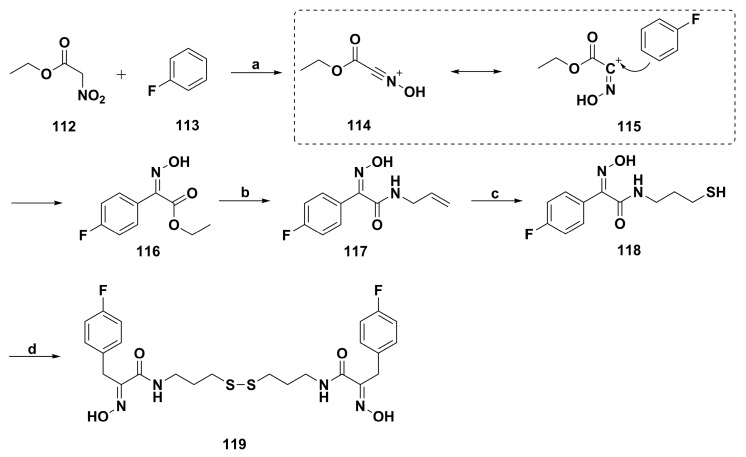
Synthesis of psammaplin A analogue **119**. Reagents and conditions: (**a**) CF_3_SO_3_H; (**b**) amine, heating neat or microwave neat; (**c**) h*ν*, R_2_-SH; (**d**) air.

Innovative synthesis of psammaplin analogues is proposed by Bertrand and coworkers through superacid, microwaves, and *S*-ene chemistry reactions as zinc-dependent HDAC inhibitors [95]. Among them, the thiol derivative **119** (Scheme 8) was five-fold more selective for HDAC 6 compared to HDAC 2. Moreover, in the bioluminescent resonance energy transfer tests, the HDAC inhibition activity of **119** confirmed the oxidative process importance in cancer cells in the environment of biomolecules being oxidized or reduced.

**Figure 10:**
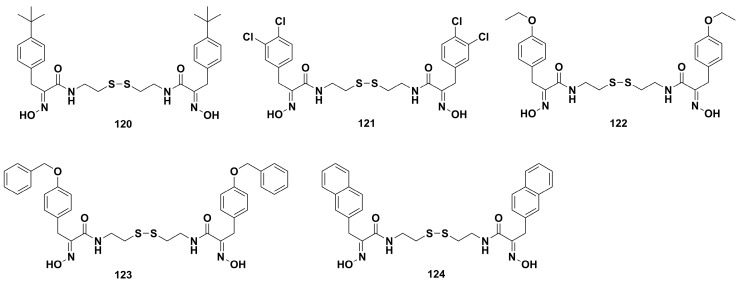
Chemical structures of the psammaplin A derivatives **120**‒**124**.

Twenty-eight derivatives of psammaplin A were prepared by Park and coworkers using a new concise approach [65]. Among them, compounds **120**–**124** (Figure 10) displayed comparable cytotoxicity to the parent compound. Especially, **120** possessed the highest antiproliferative activity against A549 cells with an IC_50_ value of 1.20 μM. Study of the structure–activity relationship revealed the disulfide bond and oxime group might be primary pharmacophores for high cytotoxicity. Furthermore, the fluorometric HDAC assay showed that **120** could inhibit the HDAC enzyme activity and enhance the expression of acetylated H3 in the A549 cells. The mechanism study showed **120** restrained the growth of A549 cells through the AKT and ERK signaling pathways. The in vivo study reconfirmed that **120** could inhibit tumor size outgrowth.

**Scheme 9:**
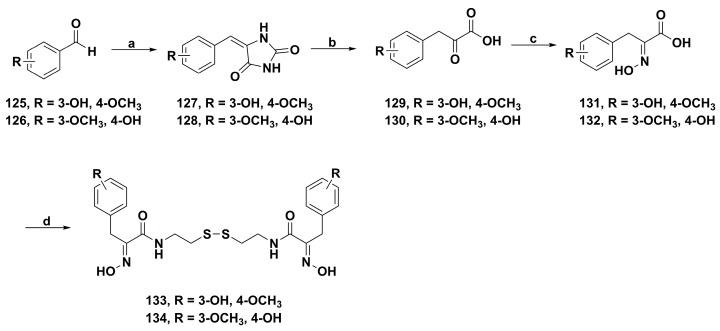
Synthesis of psammaplin A analogues **133** and **134**. Reagents and conditions: (**a**) hydantoin, ethanolamine, EtOH, H_2_O, reflux, 5 h; (**b**) NaOH, H_2_O, reflux, 12 h; (**c**) NH_2_OH·HCl, NaOH, NaHCO_3_, H_2_O, rt, overnight; (**d**) cystamine dihydrochloride, EDCI, HOBt, THF, rt, 24 h.

A collection of novel psammaplin A derivatives were synthesized by Zhao and coworkers [96]. The derivatives **133** and **134** (Scheme 9) showed potent cytotoxicity against four cancer cell lines (PC-3, MCF-7, A549, and HL-60) and better HDAC inhibition than psammaplin A. Molecular docking simulation showed that the hydrogen atom of the oxime group could interact with the active site of Asp 99 of HDAC1 via hydrogen bonding, and the hydroxyl group which could interact with Glu 203 at the entrance to the active site tunnel was optimally attached on the *para*-position of the benzene ring.

## 5. Conclusions

In summary, marine natural products, as extremely valuable resources, have become significant enablers of new drug development due to their extensive chemical variability and diverse bioactivities. Unfortunately, their in-depth study and application are restricted due to the low natural isolated yield from marine microorganisms and their poor physiological stability. Therefore, a great deal of interest has been shown in the total synthesis of marine natural products. The conventional total synthesis methods of psammaplin A were initiated from tyrosine or phenylpyruvic acid derivatives. Since 2009, improved synthesis methods have mainly used various substituted benzaldehydes as the starting materials, which made psammaplin A easier to obtain. Moreover, the pharmacological activities and structural modifications of the dimeric marine natural product psammaplin A were comprehensively summarized. Psammaplin A possesses a wide range of pharmacological activities, including antimicrobial, anticancer, antiviral, embryo development promotive, insecticidal, active chemical defense, and eryptosis-inducing activities. More importantly, it displays antibacterial and antiproliferative activities mainly through inhibiting multiple enzymes, including chitinase, HDAC, and others. To further improve its drug-like properties, a collection of psammaplin A derivatives were synthesized through structural modifications aimed to explore their potential therapeutic value and to study the structure–activity relationships. Among them, some antibacterial derivatives showed stronger antimicrobial activity against SA and MRSA via inhibiting DNA gyrase and bacterial DNA synthesis enzymes. Furthermore, the promising anticancer derivatives not only possessed stronger antiproliferative activities against various cancer cell lines, but also indicated higher HDAC 1 inhibitory activity. Finally, the structure–activity relationships revealed that the disulfide bond and the oxime moieties are indispensable pharmacophores for its bioactivities. Collectively, we hope this review will aid researchers in further studying psammaplin A.
